# Slack It to Me: Complementing LMS With Student-Centric Communications for the Millennial/Post-Millennial Student

**DOI:** 10.1177/0273475319833113

**Published:** 2019-08

**Authors:** Spencer M. Ross

**Affiliations:** 1 University of Massachusetts Lowell, Lowell, MA, USA

**Keywords:** Slack, technology in classroom, learning approaches and issues, marketing education issues, Millennial students, teamwork/projects/issues, learning approaches and issues, LMS, digital interaction

## Abstract

Past research has found that students and instructors may be disaffected with many of the most widely used learning management systems (LMS). Other research has found that Millennials and post-Millennials have come to expect open and frequent communication and technologies that facilitate greater teamwork in their business careers. The purpose of this article is to first assess the general attitudes and perceptions of widely used LMS platforms in creating an engaging student learning experience and then present and assess Slack, a business communications tool, as an LMS complement. The author finds that many of the LMS platforms present challenges for students and instructors with respect to course communications, and group communications in particular. The author also finds that Slack positively enhances students’ perceptions of the marketing class as a real-world experience, as well as enhances perceived learning outcomes from groupwork.

In early 2015, a snowstorm dumped more than 7 feet of snow on most of the northeastern United States causing major disruptions across the region. During those 2 weeks, many universities were officially closed for multiple days, while other instructors had to individually cancel or delay classes due to personal transportation issues such as delayed commuter rail schedules. Universities encouraged instructors to use learning management systems (LMS) as a stopgap measure to make up for lost student contact hours. However, LMS is not necessarily efficient in presenting course material or establishing student contact; both students (Strauss & Hill, 2007) and instructors (Schoonenboom, 2014) find instructor-centric LMS systems demotivating to use as well.

As showcased in the opening scenario, there is a need for research on both contemporary attitudes toward, and perceptions of, LMS and alternate forms of collaborative communications. If students are otherwise apathetic or antipathetic to LMS, then alternative solutions should, at a minimum, complement current systems. As Millennials (age 22-37 years) and post-Millennials (age 21 years and younger)^1^ now constitute most traditional student populations, demand has increased for tools providing an experience allowing for egalitarian communication and easy sharing of content in familiar digital interfaces. Such tools should also allow instructors to efficiently distribute course content and communications amid their other institutional obligations. While business tools and apps such as Slack hold promise for these types of solutions, they are not often presented in the literature to educators.

The purpose of this article is to first assess the general attitudes and perceptions of widely used LMS platforms in creating an engaging student learning experience and then present and assess Slack (www.slack.com), a business communications tool, as an LMS complement. A survey of students and instructors highlighted student-centric deficiencies in instructor-centric LMS. The survey found differences in perceptions between students and instructors on the effectiveness of LMSs in creating an engaging course experience. Although some participants reacted positively to the LMS platforms, many others expressed frustrations regarding the usability of their LMS platform, contributing to their disillusioned use.

Given LMS’ deficiencies, and relying on research that states that Millennials and post-Millennials have come to expect open and frequent communication (especially with supervisors) and technologies that facilitate greater teamwork in their business careers (Myers & Sadaghiani, 2010), the author introduces Slack as an LMS complement. The author argues that Slack, which has been one of the business world’s fastest growing communication tools (Honan, 2014), can allow more frictionless student–instructor communication, as well as peer-to-peer communication, especially helpful for team projects. The author supplements this argument with a pilot study providing results that contrast student perceptions of Slack with a traditional LMS platform (Blackboard) and assess its impact on perceived learning outcomes. Findings from this study suggest that Slack positively enhances students’ perceptions of the marketing class as a real-world experience, as well as enhances perceived learning outcomes from groupwork. The article concludes with implications and suggestions for future research by marketing educators using corporate tools such as Slack to make a more student-centric course experience.

## LMS as an Instructor-Centric Means of Engaging Students

Millennial and post-Millennial students are frequently disaffected by university-managed LMS such as Blackboard, Moodle, or Brightspace by D2L, often neglecting to check them, despite instructor prompts (Strauss & Hill, 2007). This is often the result of both poor LMS user interface (UI) and of students’ prior poor experiences with instructors’ LMS use. Paradoxically, LMS is designed for instructors to manage their courses, yet research by Almarashdeh (2016) using the technology adoption model found that instructor satisfaction with LMS varied, depending on the system quality, service quality, information quality, perceived usefulness, and perceived ease-of-use. On a task-level basis, Schoonenboom (2014) also used the technology adoption model, finding instructors themselves often have low intention to use LMS depending on usefulness, ease-of-use, and task. If instructors vary in how they use and engage LMS, how are students expected to maintain interest in using it?

Furthermore, many of the LMS platforms tend to be obtrusive, lacking in the contemporary UI, positive user experience (UX), and basic design principles that Millennial and post-Millennial students have come to expect from digital platforms. Importantly, most LMS platforms are either not mobile ready or not mobile friendly. Whereas instructors tend to use technology uniquely related to course instructional materials, students would rather have traditional technological tools with practical use (Buzzard, Crittenden, Crittenden, & McCarty, 2011). Additionally, while some instructors may believe that email is a more responsive means to engage students who are apathetic to LMS, a survey of undergraduate students performed by Ha, Joa, Gabay, and Kim (2016) found that overall email avoidance is a strong predictor of school email avoidance. Instructors then find it a quixotic task to get students to check course emails. It is ironic some business instructors prepare students to engage in a technologically cutting-edge workforce while they either lag in adopting new and career-relevant modes of course communication or remain dissatisfied with their institution’s technological offerings (Schoonenboom, 2014).

The result is that instructors attempting to engage classes of Millennial and post-Millennial students may find deploying LMS an inefficient and/or ineffective use of course preparation time, relative to students’ active learning engagement. For example, Strauss and Hill (2007) found that nearly half of marketing students used web-based instructional tools (e.g., email, online tests) less than once a month, despite instructors frequently investing preparation time in these tools hoping to enhance course engagement. Although instructors may tout student successes with LMS-based materials (Mehta & Kalyvaki, 2017) or even mandate student–LMS engagement (Edmunds, Willse, Arshavsky, & Dallas, 2013), students are otherwise inclined to view LMS as a course supplement rather than as a course complement.

The author conducted additional research to explore both students’ and instructors’ contemporary attitudes toward, and perceptions of, LMS. The goals of Study 1 were to determine the usage frequency of various communications platforms, to assess how LMS was perceived as contributing to the course experience, and to ascertain anonymous open-ended feedback on attributes offered by LMS platforms. Conducting this research helped ascertain where and how a tool like Slack could complement an LMS platform.

## Study 1: Student and Instructor Attitudes Toward and Perceptions of LMS

### Method

To explore both students’ and instructors’ attitudes toward and perceptions of LMS, an anonymous self-reported survey instrument was distributed on Facebook, Twitter, and LinkedIn, in exchange for an opportunity to win one of four Amazon gift codes. Students and instructors at American-based institutions were eligible to participate over a 2-week period. Of the 88 responses received, 49 usable responses were fully completed. Of the usable responses, 30 students participated, 83.3% of whom were from public universities, 76.7% were from institutions with more than 15,000 students, 73.3% were undergraduates, 70% were business majors, and 60% were born between the years 1981 and 1996, which corresponds with Pew Research’s definition of Millennial (Dimock, 2018). Another 33.3% of student participants were born between the years 1997 and 2018, which corresponds with the post-Millennial cohort.

The predominant LMS platform of student participants was Canvas (60.0%), followed by Blackboard (26.7%), Moodle (10%), and Brightspace by D2L (3.3%). Using LMS at the school was indicated by 46.7% of student participants as required for all courses, with an additional 13.3% indicating that its use is preferred for all courses. Use of LMS in three or more courses was indicated by 80% of student participants.

The remaining 19 survey participants were instructors; of these, 63.2% were from public universities, 42.1% were from institutions with more than 15,000 students, 78.9% were at the assistant professor (or equivalent) level, 63.2% were business instructors, and 52.6% were born between the years 1981 and 1996, corresponding with the Millennial cohort. Another 36.8% of participants were born between the years 1965 and 1980, which corresponds with the Gen X cohort.

The predominant LMS platform of instructor participants was Blackboard (52.6%), followed by Canvas (21.1%), Moodle (10.5%), Sakai (10.5%), and Brightspace by D2L (5.3%). Using LMS at the school was indicated as required for all courses by 15.8% of instructor participants, with an additional 10.5% indicating that its use is preferred for all courses. Use of LMS in three or more courses was indicated by 52.6% of instructor participants.

#### Instrument Measures

Participants were first asked to respond with their frequency of using 17 commonly used communications technology platforms. Additional questions surveyed participants’ typical use of email—the dominant method of modern communication.

**Figure 1.**
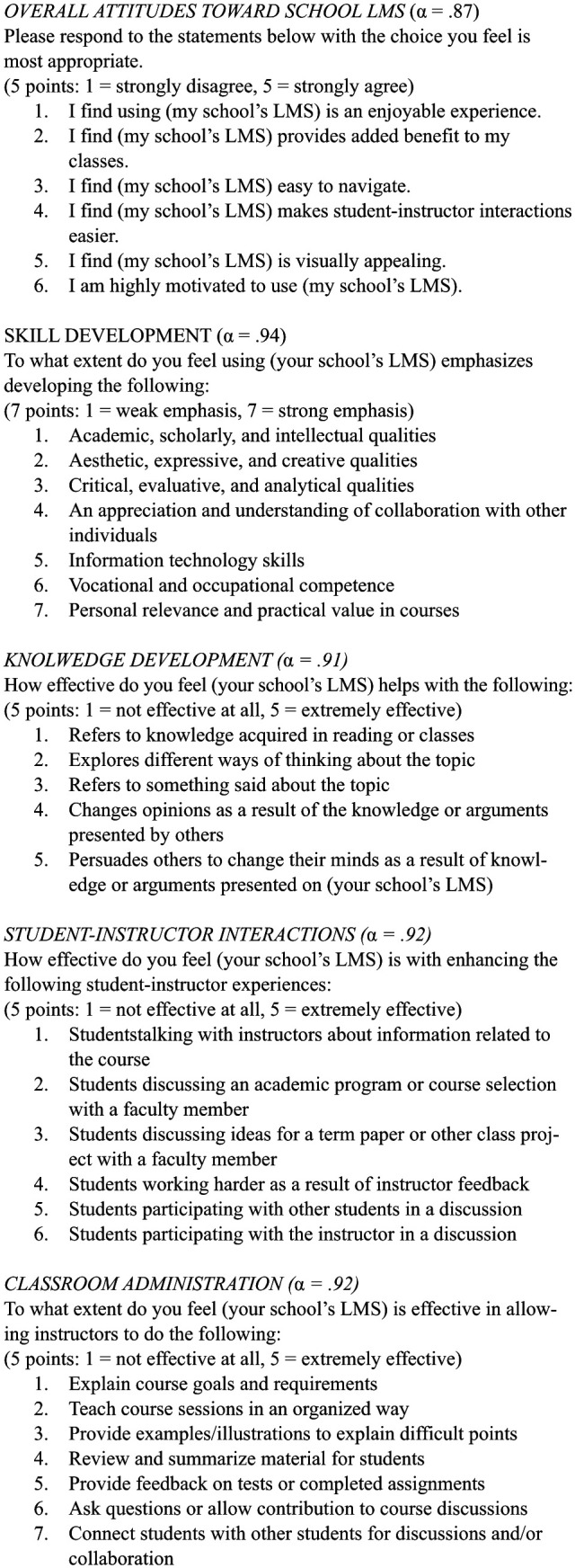
Learning management systems (LMS) survey instrument.

Next, participants were asked to rate six items pertaining to overall attitudes to their institution’s LMS on a 5-point Likert-type scale ( α = .87; 1 = *strongly disagree*, 5 = *strongly agree*; see Figure 1 for instrument items).

Elements of the College Student Experience Questionnaire (Gonyea, Kish, Kuh, Muthiah, & Thomas, 2003) were adapted around the experience of using LMS for class. As the College Student Experience Questionnaire broadly assesses students’ overall college experience, items were selected and adapted to survey the specific perceptions of LMS on the classroom experience. Participants responded to seven items assessing use of LMS to develop skills (α = .94; 1 = *weak emphasis*, 7 = *strong emphasis*), five items assessing use of LMS to facilitate knowledge development (α = .91; 1 = *not effective*, 5 = *extremely effective*), six items assessing effectiveness of LMS to foster student–instructor interaction (α = .92; 1 = *not effective*, 7 = *extremely effective*), and seven items assessing the efficacy of instructors using LMS (α = .92; 1 = *not effective*, 7 = *extremely effective*).

Finally, participants responded to open-ended questions asking them to elaborate on strengths and weaknesses of a range of 12 different LMS platform attributes. Participants were asked to provide qualitative feedback on the strengths and weaknesses of the desktop website, the mobile website, student–instructor messaging, embedded external content, private group, uploading capabilities, search functionality, mobile notifications, real-time chat/video, asynchronous chat, bulletin boards/forums, and the mobile app.

### Results

#### Use of Technology and Email

**Figure 2.**
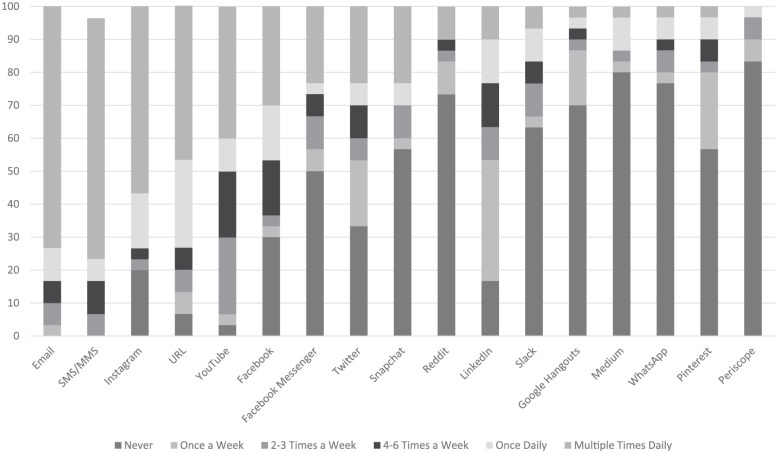
Student frequency of technology use (expressed as percentages).

Figure 2 shows the frequency of use of 17 different communications technology platforms by students. On average, 60% of student respondents check their email multiple times a day. Of student respondents, 80.3% of them send between 0 and 3 emails per day *to* instructors, but 76.7% of them receive between 0 and 3 emails *from* instructors.

**Figure 3.**
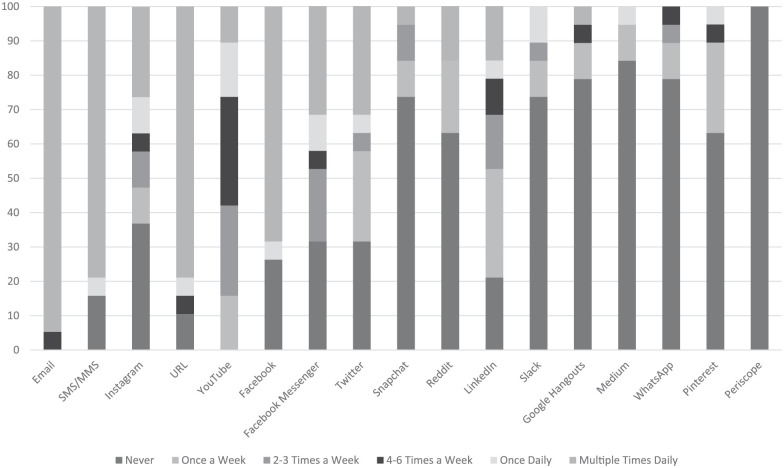
Instructor frequency of technology use (expressed as percentages).

Figure 3 shows the frequency of use of 17 different communications technology platforms by instructors. On average, 94.7% of instructor respondents also check their email multiple times a day. Of instructor respondents, 77.7% of them sent between 0 and 3 emails a day *to* students, but 66.7% of them receive between 0 and 3 emails *from* students.

#### Attitudes Toward and Perceptions of LMS

As reported in Table 1, student attitudes toward LMS were favorable (*M* =3.65, *SD* = 1.0) and did not significantly differ from instructor attitudes toward LMS (*M* = 3.24, *SD* = 0.71); *F*(1, 47) = 2.46, *ns*. Students felt that their LMS significantly emphasized skill development (*M* = 4.40, *SD* = 1.68) more than did instructors (*M* = 2.99, *SD* = 1.35); *F*(1, 47) = 9.40, *p* < .01. Students (*M* = 2.81, *SD* = 1.15) and instructors did not significantly differ in their perception of LMS effectively helping knowledge development; *F*(1, 47) = 3.21, *ns*. Students (*M* = 2.84, *SD* = 1.18) perceived LMS as less effective than did instructors (*M* = 2.24, *SD* = 0.75) at helping facilitate student–instructor interactions; *F*(1, 47) = 3.97, *p* = .05. Students found LMS to be more effective (*M* = 3.51, *SD* = 1.06) in helping classroom management than did instructors (*M* = 2.91, *SD* = 0.91); *F*(1, 47) = 4.15, *p* = .05.

**Table 1.** Mean Perceptions of LMS.

Perception	Student mean	Instructor mean	*F* test (*df* = 1.47)	*p*
Attitude toward LMS	3.65 (0.18)	3.23 (0.16)	2.46	.12
Skill development	4.40 (0.31)	2.99 (0.31)	9.40	.004
Knowledge development	2.81 (0.21)	2.25 (0.20)	3.21	.08
Student–instructor interactions	2.84 (0.22)	2.24 (0.17)	3.97	.05
Classroom administration	3.51 (0.19)	2.91 (0.21)	4.15	.05

*Note*. Standard errors in parentheses. LMS = learning management system; *df* = degrees of freedom.

#### Open-Ended Feedback on LMS Functionality

Both students and instructors provided a total of 5,500 words of open-ended feedback on the strengths and weaknesses of LMS attributes. The qualitative feedback on LMS platforms was diverse, particularly across LMS platforms and student and instructor needs.

##### Desktop compatibility

Despite LMS platforms varying in their UIs, all users expected a consistent desktop experience to serve as a foundation for the course. The desktop experience was often the dominant method of LMS engagement, and as a result, a positive UX was indicated as of paramount interest. Certain respondents indicated that their LMS performed better on desktop than on mobile web or app, which was important for both students and instructors to effectively work on the LMS. As one student described,

I have noticed that Canvas looks the same on my personal MacBook as it does on a Windows computer on campus, so that’s helpful, but by no means a huge deal. I think the navigation of any LMS should look the same on other devices, so [desktop compatibility] isn’t an impressive feature to me. It works on web. That’s good.

However, this experience stood in contrast with another student’s experience with Blackboard:

The design is terrible. In part, I feel that is because [Blackboard] includes features that work for a small, 5-person seminar as well as features supporting a 600-person lecture and yet more features for online and blended format courses. The needs of instructional format differ, and [Blackboard] ends up being bloatware that is noisy and poorly designed for all formats.

##### Mobile web compatibility

The mobile web experience was varied, depending on the LMS platform. For instance, respondents using Canvas liked the ability students could check due dates and announcements from their phone, while other platforms elicited negative responses. One such instructor responded about their LMS, “It’s garbage and impossible to navigate to different sections within a course.” The respondents’ expectation of an LMS on mobile web was to have an easy-to-use interface, yet many of the LMS mobile web options ended up frustrating both students and instructors.

##### Student–instructor messaging

Students were used to sending and receiving email as the quickest way to message instructors and instructors largely responded in kind. However, some students expressed dismay with their email correspondences with instructors. One student indicated, “Email is a challenging way to ask questions and receive answers. The timeframes are not always helpful, and it is easy to make an unclear statement.”

Canvas LMS demonstrated a middle ground, with students and instructors readily able to use the LMS messaging features as they saw fit. Both students and instructors felt this was convenient and fast. One instructor described their experience with the Canvas messaging feature:

I like that students can send me a message in Canvas and I receive an email that I can respond to directly that is sent back to their Canvas inbox. It saves a step and is clearly a step above Blackboard.

This experience significantly contrasted with the messaging experience on other LMS platforms, particularly Blackboard. As one instructor wrote, “No one sees my in-Blackboard emails. I use announcements. But I think they are largely ignored by students who don’t check their regular email.” Another instructor responded with similar frustrations:

Announcements are my only tool for communicating with a large class. But unless I offer extra credit in the headline, I swear they don’t click. I feel like I have to sell my emails. I’m officially a clickbait provider for disseminating key class info via Blackboard.

While instructors look for ways to improve communications with students, most LMS systems do not provide an adequate means of doing so. One student claimed not to have previously seen any messages from an instructor in their LMS, yet expressed interest in such a messaging feature.

I’ve never received personal messages like this in an LMS and it’s nice to have direct communication with my instructor outside of in-person contact or through email.

##### Embedded content

Both students and instructors found that this feature positively contributed to their UX. As one student described:

I think [embedded content] is necessary as some information is not available anywhere else, other than on [an instructor’s] website. Directing to other means is something that is well-crafted through Brightspace by D2L.

Students liked that they could get and view other course content easily, while instructors also liked that they could embed external links and content such as YouTube videos.

##### Private groups

Some instructors liked the ability to create private groups for grading and messaging, yet both students and instructors voiced negative sentiments about this feature. Students and instructors both recognized that students often did not use the private group feature to collaborate, even when instructors took the time to organize groups with it. Students and instructors both acknowledged that students used alternate messaging apps to communicate with each other on their phones instead of the LMS platforms.

##### Uploading content

Students and instructors found that this feature was helpful. One instructor wrote,

I create most of my materials, or I provide links and PDFs of industry resources, so I can’t just send students to a publisher website. Being able to make this material easily available to my students makes the class experience better for me and the students.

However, some students and instructors expressed limitations in their school’s LMS uploads. For some platforms, students had inconsistent experiences with using Turnitin. Instructors also found that the upload interface and navigation were cumbersome. One instructor explained their experience as follows:

Canvas tries to be all things to all people, meaning there are multiple ways to do the same thing, but it also creates problems deciding how best to do something. We previously used Blackboard. While it was less flexible, it was ultimately easier to figure out.

##### Search functionality

Several students and instructors were unaware such a feature existed. Of the few respondents who were aware of search functionality, they provided a mixed assessment of its performance.

##### Mobile notifications

Students tended to like the ability to receive mobile notifications, although some students felt notifications were overly delayed or provided redundant information. Some instructors found mobile notifications distracting for themselves and could not comprehend why students would want them. In contrast, one instructor found such a notification feature to be beneficial, but wanted their students to have more granular control over them:

I am generally happy to let students engage with material at their own pace. I do not expect daily attention/discussion from the typical student. It would be nice for these to be opt-in, so that students wanting to participate in extra-class conversation could do so without it being a burden to learners who want exposure and enrichment but not a 24/7 commitment to the topic.

##### Real-time chat/video

Most students and instructors were unaware of this being an available feature, while a couple otherwise used Zoom meeting to meet their needs.

##### Asynchronous chat

The ability to leave chat messages at one time and have them responded to later was more positively responded to by students than by instructors. One student indicated awareness that instructors prefer email instead:

Professors do not prefer this method of contact, so students are less inclined to use it. If the [asynchronous chat] feature better grabbed the attention of professors, students would use it more often.

However, students found the asynchronous chat feature highly beneficial, as stated by one student who found it attractive to solicit quick responses at their own convenience:

We needed to make groups for a project. I did not know many students in class. I found it helpful to use Canvas chat to create a team. I got quick responses because the messages got passed through to the student’s email. Our last LMS (Blackboard) did not do this and since no one checks that inbox, messages would go unanswered.

##### Bulletin boards/forums

While the concept of forums was generally responded to favorably, their usability across LMS platforms was questionable. Students did not find forums visually appealing, and instructors otherwise found them pointless for face-to-face classes. Several students begrudgingly used forums for assignments—neatly expressed by one student’s displeasure, “there’s no reason to use [forums] unless the online class requires discussion boards and, in those cases, there’s no benefit from doing so. It’s just for the grade.”

##### Mobile app

Some of the LMS platforms have a mobile app, allowing increased access and convenience to the LMS platform. Students were interested in using such an app; however, they wanted the app to be compatible with various devices. While the Canvas app was particularly regarded as a more usable app, students generally found that mobile apps had significant limitations in functionality, features, and compatibility. As a result, many students must use either mobile web or return to the desktop to effectively use the LMS platform. While some instructors were aware of students using mobile LMS apps, they did not necessarily know how they would otherwise use it for their own instructional purposes. In contrast, a couple of instructors lamented either a poor or nonexistent LMS app. This sentiment was succinctly indicated by one instructor:

Make an app. Please, dear god.

### Discussion

The results of this study shed light on several issues with LMS. Although students and instructors feel that LMS positively contributes to the course experience, the UI is often tied to the usability of the LMS. As such, the use of LMS platforms broadly leaves much to be desired by students *and* instructors. Despite platforms such as Canvas eliciting more positive sentiments, students generally tended to dislike both the UI and UX of other dominant LMS platforms. Instructors provided similar critiques of LMS platforms, especially Blackboard. Overall, the same LMS feature attributes do not translate to consistent experiences across platforms.

Aside from the need for stronger mobile web and mobile app development, two additional attributes may guide opportunities for new technology to complement LMS. First, both students and instructors acknowledge email as a standard means of interacting with each other. However, the survey of communications technology indicates that both students and instructors use other messaging and social media apps (particularly SMS) to communicate with nearly as much frequency as email. In contrast, a majority of LMS platforms adopted by universities tend to be both obtrusive and not mobile friendly, giving students the feeling that instructor communication must be monolithic, noninclusive, and hierarchical. Such instructor-centric communication does not adequately prepare post-Millennial students for the more transparent, collaborative, and egalitarian workplace that their Millennial counterparts have already come to expect (Myers & Sadaghiani, 2010).

Second, groupwork is often emphasized as an important element in marketing courses in preparation for business careers (Hansen, 2016; Lancellotti & Boyd, 2008). However, the use of LMS to facilitate group cohesion frustrates both students and instructors. Despite students using other tools to communicate with each other, LMS does not serve as a common platform that increases group cohesion. Research by Hodges and Repman (2011) found that standard LMS tools should be supplemented with Web 2.0 tools for instructors to reach students and improve student learning. Furthermore, Lowe and Laffey (2011) found that incorporating newer technologies, such as Twitter, to the course experience has a positive impact on student learning.

Overall, perceptions that LMS platforms provide value to the course experience are mixed. Depending on the platform, students who *do* engage in required LMS use often do so begrudgingly, as they have no alternate option; students who do *not* engage in required LMS use otherwise assume the implied risk of negative learning or grade outcomes. Universities typically have service-level agreements with LMS vendors and many instructors may not be inclined to seek out alternative contemporary platforms, at the detriment of student learning. In the next section, the author proposes Slack, a freemium business communications tool, as a solution that complements LMS through facile communications between both students and instructors as well as students and their peers.

## Slack as a Collaborative Course Communications Tool

Slack (https://www.slack.com) is a freemium business communications tool publicly released from beta in February 2014, which gained 500,000 daily active users in its first year (Honan, 2014; McCracken, 2015). Nearly 4 years following its official release, the platform registered eight million daily active users (Slack, 2018). Many team-organized companies such as *Business Insider*, eBay, Sony, Ogilvy, and Yelp started using Slack for cross-team integration, and some companies offering Slack in the workplace have also used it as a hiring perk geared toward attracting Millennial and post-Millennial interns and employees (Zax, 2014). Furthermore, instructors in other disciplines at other universities reportedly use Slack to engage Millennials and post-Millennials with a digital-first mind-set. In the academic context, Pappano (2018) discussed a journalism professor at the Ohio State University who uses Slack to post assignments while Kole de Peralta & Robey (2018) also described adopting Slack in their history courses at Idaho State University.

Critical to adopting a platform for student learning is the ability for an instructor to attract student “buy-in” by demonstrating the platform’s value, rather than allowing students to perceive new apps as “yet another download.” Introducing a new tool into students’ course technology repertoire should deliver value to students, be it through course usage or learning outcomes (Clarke, Flaherty, & Mottner, 2001). As a business communications and productivity tool, Slack’s growth as a platform and its potential for use in students’ eventual career contexts makes it an appropriate tool for the contemporary marketing instructor to drive student-centric communication with Millennial and post-Millennial students. Indeed, many of the feature attributes described in the following section give instructors the potential to create a “stickier” platform than LMS and, therefore, a richer course experience with active engagement (Aherne, 2016). Table 2 highlights some of the key differences in feature attributes between the most commonly used LMS platforms and Slack (GetApp, 2018).

**Table 2.** Comparison of Key LMS Platform Attributes.

Attribute	Slack	Blackboard	Moodle	Canvas
Web-based	×	×	×	×
Phone-app	×	×	×	×
Google Play/App Store rating (out of 5)^a^	4.4/4.2	3.6/4.6	3.5/2.4	4.5/3.9
University IT support		×	×	×
Pricing model	Freemium	University sponsored	University sponsored	University sponsored
Single sign-on		×	×	×
API	×			×
Third-party integration	×	× (select applications)		×
Mobile integration	×			
Mobile notifications	×			x
Desktop notifications	×			×
Real-time chat	×	×	×	×
Asynchronous chat	×			
Collaborative workspace	×	×		×
Collaborations with external terms	×			
Use of emoji/reactions	×			
Robust content search	×			
Video conferencing	×	×		×
Integration with cloud services	×			
Integration with external collaboration tools	×			
Commenting	×			
Threaded forums		×	×	×
Threaded discussion	×			
Tagging/@mentioning users	×			
Direct messaging	×			
Private group messaging	×			
Assessment management		×	×	×
Class assessment		×	×	×
Polling	× (using third-party API)	x		
Attendance management	× (using third-party API)	×	×	×
Participation management		×	×	×
Class scheduling		×	×	
Lecture mode		×		
Whiteboard text editing		×		
Course import/export		×	×	×

*Note*. Adapted from GETAPP (2018). LMS = learning management system; IT = information technology; API = application programming interface.

a As of June 2018.

## Feature Attributes of the Slack Platform

**Figure 4.**
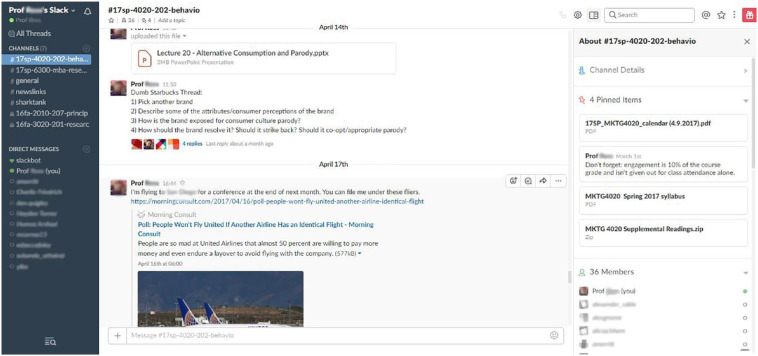
Slack user interface on web client.

As a contemporary communications platform, Slack’s various feature attributes empower students to communicate *with* the instructor, not *to* the instructor, and vice versa. With Slack, instructors can shape student expectations of the platform by reinforcing its primary function as a business tool-modified-for-educational use. Furthermore, Slack fosters increased student-centric communications among students (rather than relying on email, SMS, or social media), as well as with the instructor, breaking down power differentials and making the course more inclusive and egalitarian. This alleviates student fears that instructors may require students to merge online learning and social personae and subsequently encroach on those online spaces (Jones, Blackey, Fitzgibbon, & Chew, 2010; Tess, 2013). Following the corporate lexicon, registering for Slack merely involves the instructor creating a “workspace” as the unit of organization; the instructor becomes the workspace administrator, with the ability to modify all workspace settings. Below are several of Slack’s key attributes that make it a suitable platform for the course-as-workspace and the instructor as an active, yet equal, participant in the course experience (e.g., Hernandez, 2002). Figure 4 features a screenshot of the Slack UI on the web.

### Cross-Platform Convenience

Given the ubiquity of students with smartphones (85% of college students), combined with the increased adoption of tablets (52% of college students), student expectations of integrating traditional course content with mobile content has followed suit (Pearson, 2015). A significant drawback to LMS platforms is their lack of availability as mobile apps, as well as their poorly received web-based UI/UX (both desktop and mobile). An e-Literate/LISTedTECH report of LMS market shares find that Blackboard (28%), Moodle (25%), Canvas (21%), and Brightspace by D2L (13%) constitute 87% of the market (Hill, 2017), yet three of these four platforms do not have a robust mobile UI. Content and discussion posted on LMS is therefore regarded by students as supplementary, rather than complementary, to the course.

Slack resolves both the device and operating system issues for marketing instructors, as it is a device-agnostic, cross-platform communication solution. Slack is available for web (https://www.slack.com), iOS (https://itunes.apple.com/us/app/slack-business-communication-for-teams/id618783545), Android (https://play.google.com/store/apps/details?id=com.Slack), and Windows Phone (https://www.microsoft.com/en-us/store/p/slack-beta/9nblggh1jj9h).^2^ Both the web and mobile versions offer push notifications that allow both students and instructors to be notified on their device whenever new messages appear. While the notification settings provide a granular level of control so users need not be notified for every message, nor all times of day, instructors are welcome to set their own expectations with students of how both they and their students will be using the platform, consistent with personal pedagogical styles. Slack’s notifications contrast with LMS’ messaging tools, which rely on student pull of information or emails that research indicates students may otherwise ignore (Ha et al., 2016). The result with Slack is that both instructors and students can take their course communications and content anywhere on any device on any operating system.

### Asynchronous/Real-Time Communications Channels for Greater Collaboration

Research by Northey, Bucic, Chylinski, and Govind (2015) found that asynchronous student engagement has a positive effect on student outcomes. However, the asynchronicity of most LMS systems is limited, failing to motivate student engagement. Even if engagement is mandatory for a course (e.g., Edmunds et al., 2013), both the UX and UI of LMS bulletin boards make discussions difficult, relative to contemporary communications platforms Millennial and post-Millennial students are familiar with. Bulletin board UX is particularly conducive to students waiting until deadline to rush out postings; instead of having authentic or extemporaneous discussions about subject matter, bulletin board threads end up disjointed in subject and tone, particularly as students feel put off by the LMS medium.

Instead, Slack offers hybridized asynchronous/real-time communication in the form of “channels.” These channels are akin to always-on chatrooms, featuring a constantly running “dialogue.” One option for using channels is to have a public channel for each course section in an instructor’s Slack workspace. Students in multiple courses with the same instructor may then be able to get information for each course by joining the appropriate course channels (this can apply across semesters as well, building out a personal student social network of sorts). With the diversity of student–instructor scheduling, students and instructors can leave questions in the channels that can be responded at the convenience of the other parties, while also leaving options open for live chat and discussion when necessary.

Faranda (2015) found that out-of-class communications help improve instructor service performance. Slack allows more freedom for “virtual office hours” on an ad hoc basis and for the addition of course content at any time, giving instructors the ability to improve out-of-class communications without being intrusive in students’ lives. For example, if a student needs to miss a class or an instructor’s subway train is delayed, they could communicate with one or more people in the Slack workspace in real time, via mobile device, without losing contact hours. In another example that benefits students, Slack may help instructors provide student groups with ad hoc, afterhours, end-of-semester project guidance in a casual online context. Furthermore, integrations with Skype, Google Hangouts, or Zoom allows for embedded video communications. Since the platform allows the class to be “always on,” students may initially be intimidated by the concept, therefore instructors may wish to set expectations with students in advance, outlining why, when, and how the platform will be used by the instructor. Notifications can be modified at a granular level, with the availability to schedule “do-not-disturb” times.

Additionally, channels can be created either privately or publicly. This allows students to create their own private channels for tasks such as groupwork and/or team projects (instructors may wish to ask students to add them to private groups, if they so desire to monitor group collaboration). Research by Hansen (2016) found greater cohesion in online teams versus traditional teams; using Slack as the underlying course communications backbone allows all group members to have a common collaboration tool. Rather than necessitating emails, group SMS chats, or social media groups to set up in-person meetings for collaboration, Slack allows asynchronous/real-time group collaboration without excuse. The common platform can therefore benefit both traditional and online learning classes. Slack allows private direct messaging between users, akin to that on other social media platforms or group SMS chats. This also allows for email-like correspondence between parties, without the formality of email.^3^ Slack’s asynchronous/real-time interface is consistent across public and private channels as well as in private direct and group messaging. In September 2017, Slack introduced a shared-channels feature that allows collaborations between members of multiple Slack workspaces; this would allow students in service-based learning contexts to collaborate directly with Slack-participating clients.

As depicted in Figure 4, within Slack’s communication environment are many of the basic features of other social networks, such as Facebook Messenger, Twitter, Instagram, and Google Hangouts (“Gchat”)—familiar to Millennial and post-Millennial students on social media sites. These features range from friend lists that allow students to check the online availability of other students, to the “tagging/mentions” of other students by username, to the use of emoji and reactions. When a user tags another user using the @ sign, it sends a push notification to the said user, similar to other social networks; an “@channel” tag will notify an entire channel, and so on. In early 2017, Slack also added a “Threads” feature that allows for discussion threads within the context of the channel, while not breaking the natural flow of discussion in the workspace.

### Content Integration and Productivity

As a contemporary business communications platform, Slack channels provide various ways for both students and instructors to integrate external content, such as drag-and-drop of syllabi, assignments, lecture notes/slides, posting screenshots or other pictures, and so on. By default, Slack integrates with Google Drive to facilitate file sharing and editing. However, a series of nearly 750 apps (https://slack.com/apps)—including popular business productivity apps such as Trello (project management), Skype (video calling), Evernote (note taking), and Dropbox or Microsoft OneDrive (cloud storage) have built-in integrations to Slack. For example, a file in Dropbox can be readily imported to Slack and any changes to the file in Dropbox will be propagated to other channel members opening the shared file in Slack. IFTTT (task automation) can be integrated, so that a user (e.g., instructor) can set up a bot so tweeted links using a specific Twitter hashtag are drawn into a Slack channel without students needing to leave the platform, thereby increasing its “stickiness.” Additionally, Slack’s open application programming interface (API) allows for third-party services such as Zapier to integrate virtually any other application and for instructors with more sophisticated programming skills to add their own apps.

Content posted in Slack can also be embedded within the channel. This ensures that external content is contained on the Slack platform and available to the class in real time. For example, Figure 4 demonstrates that a YouTube link shared in a Slack message generates an embedded video, so users do not need to leave the platform to watch the video. When GIFs, screenshots, or other images are shown in messages, the visual content is also embedded without requiring users to leave the platform or download the content. Again, rather than requiring bulletin board posts or emails, Slack uploads become a part of the “conversation” and are viewable across any device on any operating system. This may be particularly useful for instructors providing virtual guidance to students about how to perform calculations or use a piece of software, wherein screenshots or pictures can be embedded in the context of asynchronous/real-time conversational exchange.

Finally, both Slack messages *and* media content are entirely searchable using an internal search function with robust search options. While the conversational stream of Slack workspaces lacks the traditional hierarchical organization of an LMS, its chronological, conversational order, paired with robust search, makes it possible to find any past content across visible channels and messages. For example, a syllabus posted in a course-specific channel at the beginning of the semester can be searched for in the Slack searchbar at any time. If students are looking for a specific conversation or keyword, they can use the search function to find the relevant conversation, in context or in file uploads (which are archived on Slack’s servers). Static files and conversation fragments can also be starred/favorited and “pinned” to a channel for static reference.

### Security

Since the primary function of Slack is business use, security is paramount for the platform. The default settings encourage domain restrictions and prevent external domains from registering to a Slack workspace. For example, an instructor can restrict registration to the @student.college.edu or @college.edu domains, preventing outsiders from joining the Slack workspace. By default, noninstitutional email addresses such as Gmail, Yahoo, and Comcast, are all prevented from registering, except for the workspace administrator (instructor).^4^ However, Slack does offer the option to manually invite a student to join the workspace.

While universities typically set up LMS to work with single sign-on for institutional security, Slack offers the individual user the option of two-factor authorization. This requires users not only to enter their password to log in on unfamiliar devices but also requires a secondary passcode from an authenticator app (which requires having a device).

### Pricing

Slack is a freemium business model, meaning it has a basic free tier, followed by two paid tiers (respectively, Standard tier pricing is $6.67 and Plus tier pricing is $12.50 per month per active user) that offer additional features such as robust user metrics/analytics. The company offers an 85% discount for educational institutions on its paid pricing models (respectively, Standard pricing is $12 and Plus pricing is $22.50 per annum per educational user).^5^ The different pricing tiers increase the number of external app/service integrations, unlimited searchable messaging, unlimited file space, guest access, and so on. The differential features in the pricing plans are high level with respect to the workspace itself and, therefore, cannot apply to select individual accounts. For example, if the instructor has chosen a paid plan, all users on the plan would be paid accounts; if the instructor has chosen a free plan, all users would be free accounts. As paying per user can be costly, particularly if instructors decide to use the platform to build and maintain a singular, personal teaching workspace over the long term, most instructors will likely find the free tier sufficient for their purposes.

## Study 2: Contrasting Students’ Perceived Learning in Slack Versus LMS

Study 1 found perceptions of LMS generally mixed, depending on the platform being used. The dominant LMS platforms provided opportunities for student-centric communications that improved student–instructor out-of-class interactions, as well as better facilitated peer-to-peer communications for areas such as groupwork. Furthermore, prior research by Clarke et al. (2001) on the value of new technology in the classroom demonstrated students’ increased interest in the technology if it had practical relevancy such as real-world use in business careers. This presents an opportunity for Slack to be used with students as a course complement to the LMS provided by the school.

Pilot data were collected on the perceived effects of Slack on students’ learning. Data were collected following a Slack implementation across two Marketing Principles sections consisting of 82 students at a large northeastern U.S. university. This implementation was done during the author’s first semester as a faculty member at the university, where students were previously unaware of Slack, as well as the author’s use of it. Registration on the platform was mandatory by the professor, as it was described to students that Slack would entirely replace both email as the primary means of communication for the course and the Blackboard LMS as the primary distribution of course materials. Basic analytics provided by Slack’s free pricing tier demonstrated that more than 4,300 messages were sent across the platform over the course of the semester. Of these messages, 23% were sent through publicly available channels, while 43% were sent in private channels and 33% were sent in private direct messages. The course used more than 300 megabytes of free storage space for 272 files.

At the end of the semester, the author asked for voluntary participation in a survey of attitudes and behaviors in exchange for course credit. Across both course sections, 44 students (54% response rate) responded to a self-reported survey that assessed both their use of instructional technologies and their attitudes and perceptions of both Slack and Blackboard. The mean age of the participating sample was 21.1 years (*SD* = 3.0), and was 64% male, 100% full-time student, 62% Junior grade-level, 64% employed part-time, and 64% felt they would receive a grade in the B range. All participants had prior experience with Blackboard but no prior experience with Slack.

### Assessing Students’ Use of Existing Course Technology

Participants were given the Young, Klemz, and Murphy (2003) scale of course technology adoption. Semantic differential scales (1 = *unfamiliar*, 7 = *familiar*) were used to measure students’ prior familiarity with a variety of course technologies. As seen in Table 3, students were most familiar with email (*M* = 6.91, *SD* = 0.29), PowerPoint (*M* = 6.69, *SD* = 0.56), and YouTube (*M* = 6.69, *SD* = 0.67) and least familiar with discussion boards (*M* = 5.09, *SD* = 1.65), Twitter (*M* = 5.53, *SD* = 1.74), and chat (*M* = 5.93, *SD* = 1.59). Students were also asked about the effectiveness of these technologies to their learning outcomes (1 = *ineffective*, 7 = *effective*). Of the technologies, students perceived PowerPoint (*M* = 6.22, *SD* = 0.88) and Blackboard (*M* = 5.62, *SD* = 1.56) as most effective, while Twitter (*M* = 2.98, *SD* = 1.60) and chat (*M* = 3.67, *SD* = 1.91) were considered least effective.

**Table 3.** Student Use of Technology.

Technology	Mean familiarity (*n* = 44)	*SD* familiarity	Mean perceived effectiveness (*n* = 44)	*SD* perceived effectiveness
PowerPoint	6.69	0.56	6.22	0.88
Blackboard	6.38	0.96	5.62	1.56
Email	6.91	0.29	4.98	1.84
YouTube	6.69	0.67	4.87	1.93
SMS/MMS	6.31	1.61	4.67	1.58
Bulletin/discussion boards	5.09	1.65	4.56	1.84
Chat	5.93	1.59	3.67	1.91
Facebook	6.47	1.27	3.07	1.75
Twitter	5.53	1.74	2.98	1.60

### Contrasting Student Attitudes Toward Slack and LMS

Participants were given a modified version of the items used in the survey by Rinaldo, Tapp, and Laverie (2011) that previously assessed the effect of Twitter on perceived student outcomes. An eight-item scale measured how students felt about the use of Slack/Blackboard as a course tool, about the role of Slack/Blackboard in career preparation, and about the role of Slack/Blackboard to achieve traditional education goals. All responses were based on 7-point scales (1 = *strongly disagree*, 7 = *strongly agree*). Use of these items for both Slack (α = .95) and Blackboard (α = .92) demonstrated high reliability.

As demonstrated in Table 4, student perceptions of the tools’ effect on learning outcomes differed on several items. Students felt Slack (*M*_Slack_ = 4.80) contributed significantly more to creating a real-world experience than Blackboard (*M*_LMS_ = 3.67, *t* = 1.13, *p* < .05). Additionally, students felt that Slack (*M*_Slack_ = 4.60) helped them more than Blackboard (*M*_LMS_ = 4.38) to become a more competent marketer (*t* = 0.58, *p* < .05).

**Table 4.** Student Evaluations of Perceived Platform Outcomes.

Using this tech helps/helped:	Slack	Blackboard	Difference	*t* test (*df* = 44)	Cohen’s *d*
Produce a high level of involvement in the course	5.13	4.91	0.22	0.61	.09
Helps understand the course material	5.11	5.02	0.09	0.22	.03
Helps me learn material better	4.98	4.71	0.27	0.69	.10
Aids in achieving overall satisfaction in courses	4.96	4.91	0.04	0.12	.02
Helps me achieve higher educational value	4.84	4.60	0.24	0.70	.10
Contributes to real-world experience	4.80	3.67	1.13	3.17*	.47
Helps me become a more competent marketer	4.60	4.38	0.58	2.09*	.31
Contributes to career skills	4.47	4.16	0.31	0.92	.14

* *p* < .05.

A 10-item scale adapted from Ueltschy (2001) measured students’ overall reactions to and attitudes about the use of Slack/Blackboard in the course. All responses were measured on 5-point scales (1 = *strongly disagree*, 5 = *strongly agree*). Use of these items for both Slack (α = .95) and Blackboard (α = .92) demonstrated reliability.

As demonstrated in Table 5, student evaluations of using the tool in the course differed on many items. Students felt Slack (*M*_Slack_ = 3.64) was significantly more fun to use than Blackboard (*M*_LMS_ = 2.91, *t* = 3.77, *p* < .05), Slack (*M*_Slack_ = 3.47) significantly improved groupwork skills over Blackboard (*M*_LMS_ = 2.58, *t* = 4.03, *p* < .05), students felt more comfortable discussing sensitive issues via Slack (*M*_Slack_ = 3.38) than via Blackboard (*M*_LMS_ = 2.69, *t* = 3.13, *p* < .05), students felt more comfortable asking clarification questions or questions about material via Slack (*M*_Slack_ = 3.53, *M*_LMS_ = 2.87, *t* = 3.09, *p* < .05), and students were better able to understand differing viewpoints via Slack (*M*_Slack_ = 3.53, *M*_LMS_ = 3.00, *t* = 2.27, *p* < .05).

**Table 5.** Student Attitudes Toward Using Blackboard in the Course.

Attitude	Slack	Blackboard	Difference	*t* test (*df* = 44)	Cohen’s *d*
I think it’s fun to use	3.64	2.91	0.73	3.77*	.56
Integration makes the class more enjoyable	3.56	3.16	0.40	1.57	.23
Helps me seriously consider differing points of view	3.53	3.00	0.53	2.27*	.34
I feel more comfortable asking questions when I don’t understand the material or need clarification	3.53	2.87	0.67	3.09*	.46
My skill working in groups improves	3.47	2.58	0.89	4.03*	.60
I feel more comfortable discussing sensitive issues	3.38	2.69	0.69	3.13*	.47
I understand the material better	3.33	3.44	−0.11	−0.41	−.06
Did nothing to enhance the understanding I gained from the course	2.78	2.67	0.11	0.37	.06

* *p* < .05.

Participants were then asked to evaluate the importance of the Slack platform features previously discussed above on their perceived learning. All responses were based on 5-point scales (1 = *not at all important*, 5 = *extremely important*). As demonstrated in Table 6, all of Slack’s features were perceived as playing a significant role in their course learning (*p* < .01).

**Table 6.** Perceived Importance of Slack Platform Attributes.

How important was the following Slack feature to your learning?	Mean	Mean difference from neutral	*t* test (*df* = 44)	Cohen’s *d*
Web/mobile compatibility	4.00	1.00	5.65*	.84
Direct messaging with the professor	3.96	.96	5.69*	.85
Embedded content from course materials	3.93	.93	5.61*	.84
Private groups	3.89	.89	5.93*	.88
Direct messaging with peers	3.84	.84	5.55*	.83
Uploading your own content	3.78	.78	4.72*	.73
Search functionality	3.78	.78	4.91*	.70
Push notifications	3.69	.69	3.97*	.59
Real-time chat	3.67	.67	3.99*	.59
Embedded external content	3.67	.67	3.96*	.58
Using current business technology	3.62	.62	3.39*	.51
Topic channels	3.60	.60	3.54*	.53
Asynchronous chat	3.58	.58	3.60*	.54

* *p* < .01.

## Implications for Marketing Educators

Despite continued evidence in support of the role of technology in marketing courses (Northey et al., 2015; Pentina, 2010; Rinaldo et al., 2011), there remains a disconnect between the rate and type of technology adoption by instructors and students (Buzzard et al., 2011). This could potentially be the result of instructors not wishing to keep pace with technology advances, Millennial and post-Millennial interest in staying ahead of the technology curve, or an interaction between both factors that would necessitate further research. However, many of the current LMS offerings provided by colleges and universities have poor UX and UI and do not seem to contribute positively toward the development of student skills and workforce readiness, as both students and instructors are apathetic toward using them (Schoonenboom, 2014; Strauss & Hill, 2007). Study 1 demonstrated mixed perceptions of LMS contributing to the course experience, implying opportunity for improved collaborative communications. Although social media platforms are often suggested as an alternative, most of these platforms are more about “posting,” and less about collaboration, whereas Slack is a rich hybrid of email/SMS/messaging that also allows direct collaboration into the platform. Furthermore, since overall email avoidance has a positive negative relationship with school email avoidance (Ha et al., 2016), students have little motivation to deal with these technologies that instructors use to communicate course content; this also impedes student collaboration both in and out of the classroom, among each other, and with the instructor.

This article proposed that Slack offers a student-centric communications backbone for marketing educators to better engage Millennial and post-Millennial students with course content. Past research by Clarke et al. (2001) demonstrated that students perceive the real-world relevancy of technology as important to its course use. The results of a pilot study using self-reported data found that, overall, Slack helped students feel like the course contributed to their real-world experience. Furthermore, students felt the use of Slack in a course was beneficial to them, particularly when made aware that many workplaces offer the platform for internal communications and collaborations. As Slack’s primary clientele is corporate users, its continuous development has the potential to affect class use.

The study found that students perceived improved engagement with instructor/peers and that the platform’s major features were perceived as helpful to the learning environment, despite no evidence, Slack fundamentally improved students’ comprehension of materials or direct learning outcomes. Nonetheless, students felt that Slack transformed the course into a team-like atmosphere, reducing the monolithic feel of the instructor–student relationship and transforming the content into one that felt more student-centric, collaborative, and egalitarian. Since active learning and teamwork are regarded as standard learning outcomes of many courses (Hansen, 2016; Hernandez, 2002; Lancellotti & Boyd, 2008), Slack benefits students by fostering collaborative culture in and out of the classroom—similar to what students would expect and experience in the workplace.

Slack’s feature attributes as a communication platform are generally held in favorable regard. For Millennial and post-Millennial students, this is an additional benefit, since this group of students is used to communicating between peers, family, friends, coworkers, and managers on simple, frictionless platforms (Myers & Sadaghiani, 2010; Pearson, 2015). Telephone conversations, and even emails, have become antiquated forms of communications for instructors and students alike (Strauss & Hill, 2007), yet instructors have declined to foster out-of-class communications in a way that is frictionless, readily accessible to students, and career-oriented. Slack solves this by maintaining “always on” communications in a similar fashion to that offered in marketing careers. In contrast with traditional LMS platforms, these advantages and flexibilities also allow instructors to more efficiently manage their course content and communications within a platform that several firms already offer their employees.

### Implementation and Suggested Practices for Instructors

Given the openness of the Slack platform, there is a lot of flexibility in how instructors may wish to administer their workspaces. As with many modes of communications, the successful use of Slack starts with the cultural expectations and norms set forth by the instructor. The goal of this section is to provide initial suggestions of practices instructors may consider when using Slack with their courses. Instructors are otherwise encouraged to review and explore the different platform settings to find customizations that are best suited for their own needs.^6^

#### Setting up the Workspace

To use Slack to foster collaboration and community, setting up courses in a single workspace (vs. multiple workspaces) serves multiple advantages for the instructor. First, it allows the instructor to develop a workspace as a personal learning network. In using the free version of the platform, this yields the added benefit of allowing the instructor to “retain” members of the network beyond the scope of a semester course. Second, it allows both the instructor and students to use the same workspace for multiple courses, eliminating the need to log into multiple workspaces. For a student enrolled with an instructor over multiple courses (or even multiple semesters), this necessitates only a single Slack registration. Finally, it allows the instructor to divide content up by different courses across different channel topics, minimizing the amount of effort the instructor needs to invest in setting up the workspace.

On the initial registration, all members of the workspace are added to the #general channel. One recommendation for instructors is to use this channel for communications across all courses and students, including class cancellations, job opportunities, and university lecture/event announcements. Additional channels may be set up as desired for individual courses using a descriptor that students would be able to search and understand (e.g., course number/section). These course channels would function for course-specific announcements, discussions, and threads. Instructors should then let students know to search for the course channel. Additional channels can otherwise be created for specific topics of interest. For example, the author set up a #newslinks channel and modified the registration settings so new members are also added to this channel by default; the author then uses the automation tool IFTTT to pull their tweets that use a specific hashtag into the #newslinks channel for students to read and engage. Other integrations include the Simple Poll app, which can be used to create a native poll within Slack. Instructors may also wish to add team collaboration app integrations such as Dropbox, Skype, and Trello for students to use for team collaboration. A full list of searchable app integrations can be found at https://slack.com/apps.

As a workspace builds on itself from semester to semester, another recommended practice for the end of semester is for instructors to hide and archive prior course channels. This can be done by going to the Settings icon at the top of the channel and selecting “Additional Options.” Hiding a channel makes it hidden from public/searchable view; however, members in the channel can still see it in their active channels list. Hiding the preceding semester’s channel would then allow students to still have visible access to the channel and its contents. Archiving a channel hides the channel from the active channel list but retains the data for the instructor for a future point if necessary. Hiding beyond an additional semester would eliminate the channel from visible access, making the workspace less confusing to both instructor and students.

#### Student Buy-In via the Syllabus

To shape student expectations of instructor use of Slack for course content and communications, it is important that instructors adequately emphasize the use of Slack instead of email and the selective use of LMS in the course at the onset of the course. This may be done with redundancy in three different areas. First, the instructor should think to include verbiage in the syllabus that formally sets out instructor expectations of technology use; this may be included under the “required materials” section of the syllabus. An example of such verbiage may read as follows:

Slack is a business communications tool we’ll be using instead of email/text. It’s available for web/iOS/Android, has discussion threads, private group messaging, direct messaging, drag-and-drop file sharing, search, Dropbox/Google Drive integration, mobile push notifications, and gifs. ***All course communications (i.e., cancellations, announcements, discussion) and supplemental course materials (i.e., links, other readings, slides) will be posted through Slack—*NO EMAILS**.

Register with *@[schooldomain.edu]* at https://mktprof.slack.com (team name: mktprof). Join the course’s channel by clicking “CHANNELS” and searching “#4500-service-mktg” (you’ll also be automatically added to the default #general and #newslinks channels). LMS will *only* be used *for individual assignment submission and gradebook purposes.*

Second, information should be verbally presented to students on the first day of class when instructors lay out expectations for the course. The author includes four graphical PowerPoint slides in the first class presentation. Slide 1 includes a chart of Slack’s growth, a graphic (from Slack) of what it is used for in the workplace, and bulleted registration information from the syllabus verbiage. This slide emphasizes that many businesses use Slack in the workplace (as well, ad hoc Slack communities develop around interests) and points out that, in contrast with LMS tools, it is not “yet another download.” At this point, students can recognize the benefits of the platform beyond LMS, both in the class environment and in potential career and collaborative use. Slides 2 and 3 provide registration screenshots for both the web and app, demonstrating how to join channels in the workspace. This also provides a time for instructors to have their students register for Slack, join any required channels, and answer student technical questions. Slide 4 provides a screenshot reviewing the information prepopulated in the Slack workspace and pinned to channels on sign-in (syllabus, assignments, calendar, any supplemental files, any course links, a Quick Start/FAQ Google Document).

Finally, a link to a Quick Start/FAQ Google Document may be posted at the top of the course LMS for students’ reference. Although this is redundant, it allows students to have the information outside of class, as well as provides the information to late course registrants.

## Limitations and Directions for Future Research

Both studies provided here offered small sample sizes. While Study 1 featured both student and instructor respondents, the author was only able to obtain a small sample size during data collection. The student respondents were predominantly Canvas users and the instructor respondents were predominantly Blackboard users, the proportions of which did not reflect current LMS market shares (Hill, 2017). Therefore, Study 1 incorporated open-ended data as a means of supplementing the closed-response survey questions with richer data. Because of concerns from the institutional review board at the author’s university, the type and scale of Study 2 data collected was limited to self-reported survey data from a single-semester implementation over two Marketing Principles sections of 41 students each. Student studies are susceptible to nonresponse bias (Bacon, Johnson, & Stewart, 2016) and the response rate of the small sample in this study is low (54%), which may have biased the self-report data in favor of students who were routinely engaged in the author’s course. However, given the author’s continued use of Slack in subsequent semesters across various courses at the same institution, samples from the same student population may also be non-naive for future testing. The author’s institutional review board also limited the type of data that could be assessed; indirect measures of learning outcomes and student attitudes toward the platforms/features were used, rather than direct measures of learning outcomes and assessment (Bacon, 2016; Elbeck & Bacon, 2015). Despite sampling limitations, difference testing found moderate effect sizes for significant results. Future research on Slack that robustly addresses both limitations may be taken up by other instructors who adopt the platform and compare it with other active learning platforms—particularly in a controlled, wide-scale experimental design.

Currently, there are two disadvantages to using the Slack platform, both of which may stem from a conflict between Slack’s use of an open API for business integration and the closed-circuit systems of LMS.^7^ This may be a result of the Family Educational Rights and Privacy Act (FERPA) in the United States providing privacy protection for student records. First, Slack does not provide any assignment/grade management tools akin to those found on most LMS platforms. Second, there is no means to integrate Slack with a free external gradebook. LearnDash, a WordPress LMS, has a Slack integration that is a premium add-on (https://www.learndash.com/add-on/slack); however, it requires the college or university to use the WordPress LMS. As a result, both issues may still require a bare minimum use of LMS for FERPA-compliant assignment/grade management. At a future point in time, LMS platforms may resolve FERPA issues with open API, allowing integration with external providers such as Slack and offering a wider array of options for FERPA-compliant external assignment and gradebook integration.

In summary, the goal of this article was to highlight deficiencies in using instructor-centric LMS communications with Millennial and post-Millennial students, instead promoting Slack, a communications platform currently used in business, as an alternative that more actively engages students. There are open Slack communities that use the platform beyond the scope of the class workplace (e.g., https://slofile.com/category/Marketing) and can readily be switched within the app/web, yielding a potential value-add for students, beyond the workplace. LMS offers none of the above and is solely relegated to higher education use.

That Slack is under continuous development creates opportunity for instructors to continuously innovate how to effectively and efficiently use Slack for collaborative student communications. For example, the results of the second empirical study are limited to the best practices that have evolved from the author’s own courses and pedagogical idiosyncrasies. Different pedagogical styles may lead to more passive or more aggressive approaches to using Slack to engage students. Furthermore, increased use and familiarity of the platform has the potential for experimenting with the platform attributes over the long term in ensuring that courses meet assurance of learning requirements (e.g., Loughry, Ohland, & Woehr, 2014). Since the author has continued using the platform since the original data collection, it is possible that the author’s refined use of the platform may have had direct, substantive impact on student learning outcomes and course successes. Future research on the use of Slack in marketing courses, and at different curricular levels, may address differences in instructional use on learning outcomes and course successes.

## Notes

1. Pew Research defines “Millennial” as anyone born between 1981 and 1996 and “post-Millennial” as anyone born between 1997 and the present (Dimock, 2018).

2. Windows and Mac desktop clients are available; however, they are merely wrappers for the web interface.

3. While the author appreciates some instructors lament the loss of formal business communication, “flat” egalitarian communication is now a practical reality in business—particularly for Millennials and post-Millennials (Myers & Sadaghiani, 2010).

4. There are complex workarounds where the workspace administrator can link external services such as Heroku using the Slack API so students may self-register using personal email accounts, however they are omitted here, as most instructors would be inclined to close their Slack workspace from personal registrations.

5. Pricing as of June 2018. Visit https://slack.com/pricing for current pricing details.

6. An example Quick Start/FAQ Google Doc created by the author can be found at https://bit.ly/SlackJMEFAQ.

7. Of the four major LMS platforms, Canvas is currently the only one that has an API with future potential for developing Slack integrations.
